# The Epidemiological Patterns of Hepatitis C in Lithuania: Changes in Surveillance from 2005 to 2018

**DOI:** 10.3390/medicina57101120

**Published:** 2021-10-17

**Authors:** Samanta Grubyte, Jurgita Urboniene, Laura Nedzinskiene, Ligita Jancoriene

**Affiliations:** 1 Department of Public Health, Institute of Health Sciences, Faculty of Medicine, Vilnius University, 03101 Vilnius, Lithuania; 2 Clinic of Infectious Diseases and Dermatovenerology, Institute of Clinical Medicine, Faculty of Medicine, Vilnius University, 03101 Vilnius, Lithuania; Ligita.Jancoriene@santa.lt; 3 Center of Infectious Diseases, Vilnius University Hospital Santaros Klinikos, 08410 Vilnius, Lithuania; Jurgita.Urboniene@santa.lt; 4 Department of Anatomy, Histology and Anthropology, Institute of Biomedical Sciences, Faculty of Medicine, Vilnius University, 03101 Vilnius, Lithuania; laura.nedzinskiene@mf.vu.lt

**Keywords:** viral hepatitis C, infectious disease epidemiology, hepatitis C prevention

## Abstract

*Background and Objectives*: Viral hepatitis C infection is an important public health concern worldwide because it is one of the major global causes of death and morbidity. The early identifi-cation of infected people, together with the provision of proper treatment, are the key strategies used in preventing HCV infection. However, data regarding the epidemiological patterns of hep-atitis C in Lithuania are limited. The aim of this research was to evaluate trends in acute HCV cases registered via the national surveillance system in Lithuania during 2005–2018. *Materials and Methods*: Incidence rates were calculated for data, stratified by gender, age group (0–24, 25–54, 55–74, and 75+), place of residence (urban or rural), and the Lithuanian county where the case was recorded. The crude incidence rate of hepatitis C was defined as the number of new infec-tions per 100,000 people during a one-year time period. A linear regression was applied to evalu-ate IR trends during the period of 2005–2018. *Results*: From 2005 to 2018, 572 cases of acute hepa-titis C were registered, and the incidence rate ranged from 2.03 cases per 100,000 people in 2005 to 0.55 in 2016. A statistically significant decreasing trend in the incidence rate was found during the study period (*p* < 0.0001). Almost 63% of the acute HCV infections recorded via the national surveillance system were in individuals aged from 25 to 54 years. *Conclusions*: Being male, aged between 25 and 34 years, and living in the city might be important factors for understanding the epidemiological patterns of HCV in Lithuania. Although the number of HCV cases recorded in Lithuania is decreasing, our country has one of the highest IRs compared to other European countries. This shows that a new approach to hepatitis C virus screening strategies is needed.

## 1. Introduction

Hepatitis C virus (HCV) infection is one of the major global causes of death and morbidity, and so it remains an important public health concern in Europe. Lithuania is no exception [1]. The highest burden of HCV infection was estimated to reside in the eastern Mediterranean region and the European region in 2015 (both with about 62 cases per 100,000 people). More than half of asymptomatic acute HCV cases tend to evolve to chronic cases. Currently, 14 million people chronically infected with HCV live in the European region, with a significant risk of developing cirrhosis within 20 years [2,3]. It has even been estimated that while the incidence of HCV infection seems to be decreasing, mortality secondarily related to HCV infection will continue to increase over the next 20 years [4].

Although HCV case definition, screening, and diagnostic strategies vary between countries, the data described above indicate that HCV infection can be insufficiently represented in official statistics. The HCV rate reported for Lithuania in the annual report of the European Centre for Disease Prevention and Control was one of the highest rates reported across Europe in 2017. Additionally, three countries—Hungary, Lithuania, and the Netherlands—only reported acute HCV cases [5]. Data from the review conducted in 2017 showed that the prevalence of anti-HCV among first-time blood donors was highest in Bulgaria, Romania, Greece, Latvia, and Lithuania [6].

No vaccines against HCV infection exist. Consequently, for primary prevention, early identification of infected people (especially asymptomatic carriers who are not aware of their condition), together with the provision of proper treatment, are the key strategies used in the prevention of HCV infection. However, routine screening is presently limited to just blood donations in Lithuania. The testing of other risk groups, such as medical personnel, members of the military, and pregnant women, is usually performed as a part of scientific research. Two main HCV screening strategies are currently implemented worldwide: the systematised screening of risk groups specific to a particular region [7,8,9] and the screening of potentially affected birth cohorts [10,11]. Currently, the potential development of a national HCV screening programme is under intense debate in Lithuania. An HCV screening strategy that mainly focuses on the screening of risk groups specific to Lithuania in the primary care centres is under consideration.

Previously, HCV infection was difficult to eradicate. With the discovery of very effective direct-acting antivirals, almost all HCV infection can be cured [12]. The WHO released the ‘2030 Agenda for Sustainable Development’ and called for international action to combat viral hepatitis, with the aim of drastically reducing the disease burden by 2030. One of the main barriers to achieving this goal indicated by the WHO is incomplete local surveillance systems [13,14,15]. To summarise, the identification of the epidemiological patterns of HCV infection and HCV-infected groups is crucial in order to establish prevention and screening strategies.

The aim of this research is to describe trends regarding acute HCV notification via the national surveillance system during 2005–2018 in terms of gender, age group, place of residence, county, where the case was recorded, and the potential source of exposure reported by the infected person. The results of this might be beneficial to the optimisation of ongoing and future HCV control strategies.

## 2. Materials and Methods

### 2.1. Surrveillance and Notification of HCV Infection in Lithuania

In Lithuania, it is mandatory for all the licensed healthcare institutions to notify regional departments of the National Public Health Centre of all individual cases of acute and chronic HCV infections. Hospitals also oversee the reporting of laboratory testing results. Once information from the healthcare instituion is sent to the local public health institution, an assigned public health specialist completes a detailed notification form including clinical manifestations and potential risk factors and behaviours. All the cases are classified and described in the centralised surveillance database. In Lithuania, acute hepatitis C has been a reportable disease since 1993.

The main sources of data for this analysis were the publicly available statistical reporting forms published monthly and annually by the Centre for Communicable Diseases and AIDS and notification information gathered from the National Public Health Centre. No informed consent was required for our analysis as the notification of HCV cases is mandatory under Lithuanian law. Permission was obtained from the Vilnius Regional Research Ethics Committee to use aggregated and anonymised information from the national surveillance database (No. 158200-17-960-464, 2017-11-07).

### 2.2. Case Definition

Acute HCV infection is usually defined as a six-month time period following exposure to the hepatitis C virus with the onset of symptoms such as jaundice and/or peak elevated serum alanine aminotransferase. Based on the European Commission’s decision of 28 April 2008 and the amending decision 2002/253/EC, which described case definitions for reporting communicable diseases to the community network under Decision No. 2119/98/EC of the European Parliament and of the Council, confirmed acute HCV cases should meet at least one of the following two laboratory criteria: (1) the detection of hepatitis C virus nucleic acid in serum; and (2) a hepatitis C virus-specific antibody response confirmed by a different antibody test [16]. HCV infection is considered to be chronic when liver inflammation continues for at least six months. When the disease persists for a long time, the development of liver cirrhosis or cancer is likely. In Lithuania, only acute hepatitis C cases were compulsorily registered until 2019.

### 2.3. Statistical Analysis

A descriptive analysis was conducted for all the acute HCV cases officially registered via the Lithuanian mandatory notification system from 2005 to 2018. Analyses comprised the proportion of cases by gender, age group, place of residence, and Lithuanian county where the case was recorded.

The crude incidence rate (IR) of hepatitis C was defined as the number of new infections per 100,000 people during a one-year time period. Specific rates were calculated for data stratified by gender, age group (0–24, 25–54, 55–74 and 75+), place of residence (urban or rural), and Lithuanian county where the case was recorded. A linear regression was applied to evaluate the IR trends, and Poisson regression was run to evaluate changes in acute HCV infection cases during period of 2005–2018. The chi-square test was used to evaluate differences in proportions between ordered groups. Differences between proportions were considered significant if *p* < 0.05.

Data regarding 203 acute HCV cases in individuals interviewed by specialists from regional public health institutions from 2010 to 2017 were used for the evaluation of the assumed source of exposure.

The annual statistics regarding the Lithuanian HCV rates by gender, age, place of residence, and county that were used as factors in the calculation of the IR were provided by Statistics Lithuania.

## 3. Results

Between January 2005 and December 2018, 572 cases of acute HCV infection were recorded among people living in Lithuania. Cases of acute hepatitis C comprised 29.8% of all the recorded cases of acute viral hepatitis in Lithuania during the research period; acute hepatitis B comprised 40.9% of all the cases; acute hepatitis A comprised 29.2%; and acute hepatitis D comprised 0.1%.

**Figure 1:**
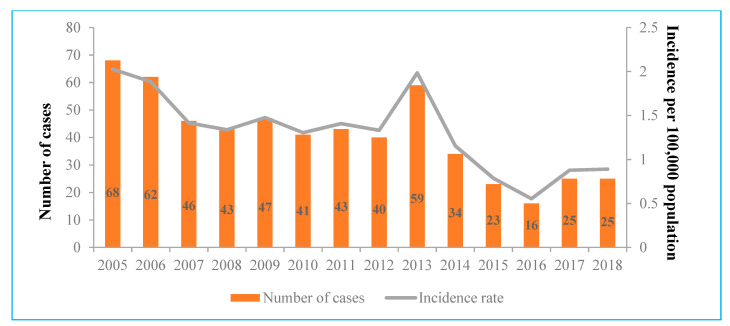
Number and incidence rates of acute hepatitis C virus cases notified via the national surveillance system, Lithuania, 2005–2018.

The IR of acute hepatitis C ranged from 2.03 cases per 100,000 people in 2005 to 0.55 in 2016. During the period from 2007 to 2012, the IR of acute cases was almost stable, with 1.3–1.5 cases per 100,000 people, before reaching a peak in 2013 (1.99 cases per 100,000 people). Every year, number of total acute HCV infection cases decreased by 7.3% (0.93, 95% CI 0.91–0.95, *p* < 0.0001) and the IR decreased by 0.08 per 100,000 people (0.08, 95% CI −0.13 to −0.04, *p* = 0.001) (Figure 1).

**Figure 2:**
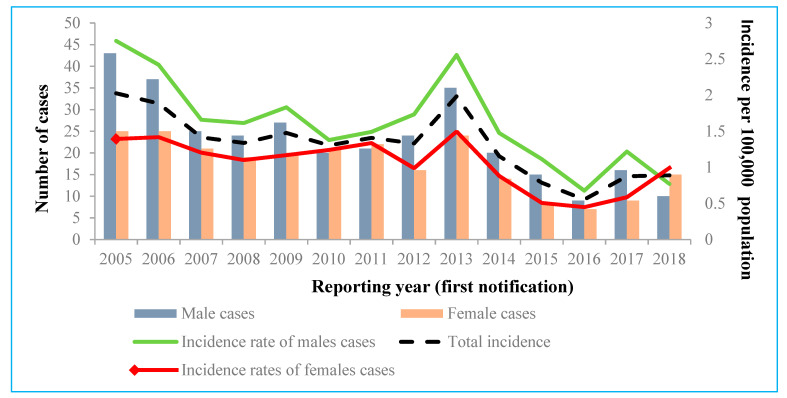
Number and incidence rates of acute hepatitis C virus cases by gender, Lithuania, 2005–2018.

During the study period, 56.9% of all the registered acute HCV cases were males. The overall IR of acute cases between 2005 and 2018 was significantly higher for males (1.65 per 100,000 people) than for females (1.07 per 100,000 people) (*p* < 0.001; OR 1.54; 95% CI 1.30 to 1.83) (Figure 2). Unexpectedly, the IR for women evaluated in 2018 (0.99 per 100,000 people) was higher than the IR for men and the total IR. Future monitoring is required, because this change in the IR can be caused by the small numbers of acute HCV cases recorded.

**Figure 3:**
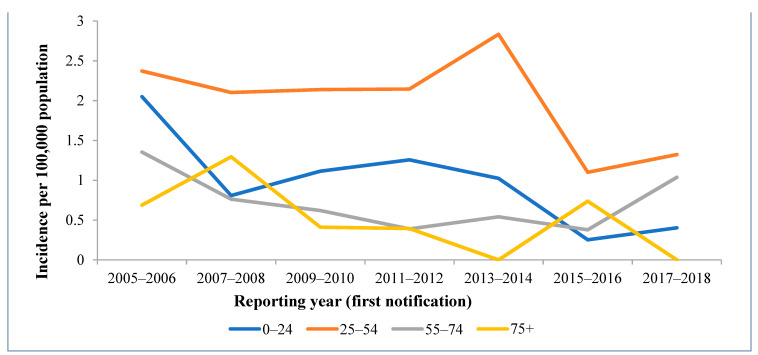
Number and incidence rates of acute hepatitis C virus cases by age group and notification period, Lithuania, 2005–2018.

As shown in Figure 3, the trend of the IR is mostly driven by people aged from 25 to 54 (62.8% of all the HCV cases); a particularly high proportion of cases were reported for people aged 25–34 (28.0%). The number of HCV infection cases was particularly low among children under 17 years old and elderly people above 75 years old. However, since 2013, the IR in people aged between 55 and 74 started to increase.

Overall, during the period of 2005–2018, the incidence of acute hepatitis C virus infection significantly decreased by 13% each year (*p* < 0.0001); the IR decreased by 0.11 per 100,000 people each year in the 0–24 age group (*p* = 0.011). The incidence rate of acute hepatitis C decreased by 7.2% in the 25–54 age group (*p* < 0.0001). A fairly high but statistically insignificant 11.2% decrease in the incidence of acute hepatitis C was seen in 75+ age group (*p* = 0.064); however, the IR decreased significantly by 0.07 per 100,000 people each year (*p* = 0.027) (Table 1 and Figure 3).

The mean annual IR of acute cases for the whole period was significantly higher for urban residents than for rural residents, with rates at 1.62 cases per 100,000 people, compared with 0.87 cases per 100,000 people, respectively (*p* < 0.001; OR 1.86; 95% CI 1.52 to 2.30).

**Figure 4:**
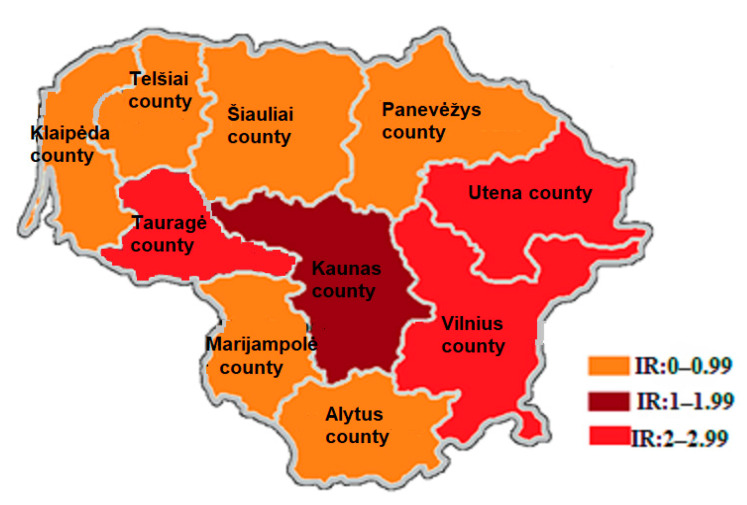
Average incidence rates of acute hepatitis C virus by the county where cases were reported, Lithuania, 2005–2018.

Of all the reported acute HCV cases, 60.1% were reported in two counties of Lithuania—Kaunas and Vilnius. However, the highest average IRs for the study period were in Utena county (2.90 cases per 100,000 people), Tauragė county (2.06 cases per 100,000 people) and Vilnius county (2.01 cases per 100,000 people) (Figure 4).

**Figure 5:**
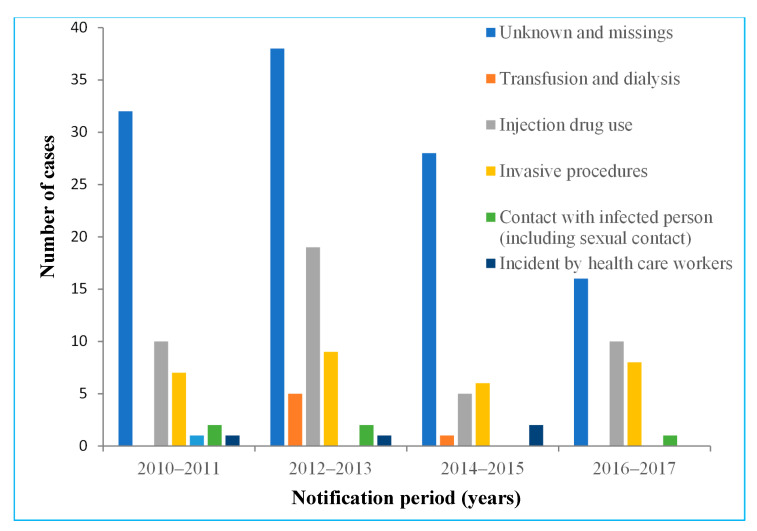
Number of acute hepatitis C virus cases by exposure and notification period, Vilnius and Kaunas counties, 2010–2017.

For the evaluation of potential exposure sources, 203 subjects from the counties of Vilnius and Kaunas were included. Only 43.8% of the subjects included in this analysis reported potential exposure. The use of injection drugs was the most commonly reported potential exposure source by the individuals within the cases studied (49.4% of cases with a reported exposure), followed by invasive procedures (including medical procedures, surgery, dental care, tattooing, or piercing). Figure 5 shows that injection drug use was steadily reported as a primary risk factor throughout the study period, with a peak of 19 cases in 2012–2013. Invasive procedures were the second most commonly reported exposure source, possibly because these procedures are the most commonly experienced and people may have been more likely to remember them. Additionally, it is important to highlight that the number of unknown or missing cases steadily decreased during the period from 2010 to 2017.

## 4. Discussion

In order to develop efficient policies and evidence-based screening strategies, data regarding the epidemiological patterns of HCV should be continuously evaluated. This article describes changes in acute HCV infection notification trends in Lithuania during the research period from 2005 to 2018.

The peaks of notification occurred in 2005 (with 2.03 cases per 100,000 people) and 2013 (with 1.99 cases per 100,000 people). If the incidence peak in 2005 can be explained by the establishment of a modern acute HCV cases registration system, it is quite difficult to understand what caused the increase in 2013. This peak in 2013 can be partly explained by the implementation of mandatory nucleic acid amplification technique (NAT) testing of Lithuanian blood donors in 2012. Although the IR of acute HCV infection was found to steadily decrease (a statistically significant downward trend is represented in Figure 1), IRs described in this survey were almost twice as high compared to the rates in counties in the European Union/European Economic Area for the period from 2012 to 2017 (Table 2). Of course, this comparison between countries can be affected by the differences between HCV diagnostic and notification systems, but it plays an important role in understanding where Lithuania stands compared to other countries.

Decreasing rates of diagnosed and reported acute HCV cases in the European region might not only indicate the positive changes in HCV epidemiology, but also point to the problems relating to early HCV diagnosis and notification. In Lithuania, as in many other EU/EEA countries, until 2019, only acute hepatitis cases were compulsorily registered. Almost three times more cases of hepatitis were registered in the first half of 2019, after the introduction of chronic hepatitis registration in Lithuania, than in the same period the previous year. As per information taken from the systematic review of hepatitis C epidemiology in Europe, Canada, and Israel, there has been no downward trend in HCV infection prevalence in Europe (except France) [17].

As per the results of our analysis, HCV infection was more commonly reported for males than for females, and this corresponds with epidemiological patterns evaluated in western and northern Europe countries [5]. This might be explained by the fact that injection drug use, which was evaluated as the the most common risk factor in our study, is much more commonly reported in men [18]. Other reported exposures remained rare overall. For example, only two people reported sexual intercourse with an infected person as a potential source of the exposure (one male and one female) (described as contact with infected person in Figure 5). However, men having sex with males has been well documented as a significant risk factor for HCV infection in the literature [18]. This exposure route of infection might be described as unknown and missing in our study, as potential risk factors were not reported for more than half of the cases.

The trend of IR for acute hepatis C virus is largely driven by people aged from 25 to 54 (Figure 3), and especially those aged 25–34. This once again might be explained by injection drug use, which was reported as the primary exposure source in our analysis. As per a study conducted in Switzerland, 70.4% of acute HCV cases in those aged from 20 to 34 potentially had experience with injection drugs [18]. Additionally, testing for HCV infection is likely to be most accessible to individuals in this age group (e.g., through the testing of pregnant women, blood donations, and testing at work), so this might be the result of the screening of various risk groups. However, a noticeable decrease in IR was observed for the 25–54 age group during the study period, and this might mean that nowadays, those with acute cases become infected later in life than in the past. Additionally, a sudden decrease in the IR for the 25–54 age group was noticed in 2015–2016 when compared to previous years. This can be explained by the fact that an extremely low number of cases related to the use of injection drugs was reported in the 2014–2015 period compared to other yearly periods. The increase in the IR in the 55–74 age group in recent years could be a coincidence, but we cannot rule out the possibility that asymptomatic cases of HCV are diagnosed late, especially within the context of the declining epidemic.

The mean annual IR of acute cases for the whole period was significantly higher for urban residents than for rural residents. This difference might be affected by the incidence peak in urban areas that started in 2013. As per the official statistics regarding trends of drug use in Lithuania, drugs are more frequently taken by residents of urban areas, so this can be a cause the spread of HCV in cities [19]. Additionally, this IR difference may indicate that rural areas deal with the bigger problem of effective screening for HCV compared to urban areas.

Injection drug use was the most commonly recorded source of exposure for acute HCV cases in Lithuania, and this reflects the general trends across Europe [5]. Anti-HCV prevalence among injection drug users has been estimated to be 67% worldwide. The recorded midpoint prevalence estimates in Europe range from 21.1% to 90.5%, with approximately half of all countries estimated to have a 60% prevalence or higher [20]. The rates at which injection drug use was reported as a potential exposure source varied through the study period. This instability can be explained by the lack of continuous screening programmes and harm reduction measures for viral hepatitis among injection drug users. Additionally, changes in the structure of potential exposures in Lithuania are highly affected by unknown or missing data (56.2% of all the analysed cases). Exposure sources commonly reported across the European region, such as sexual transmission between men who have sex with men and HIV-infected people [21,22], were not identified during our analysis. This shows that HCV screening currently available in Lithuania and the notification system might miss some significant data that could aid in the prevention of this infection in risk groups.

The identification of potential exposures to acute HCV cases in Lithuania is based on the anamnesis of the subject and their willingness to share all their personal information. Therefore, important aspects of potential risk factors might not be covered by the official statistics (intentionally or not). This indicates a lack of an effective national screening programme for HCV. Even the WHO has agreed that the public health threat of viral hepatitis has long been underestimated and recently the ‘2030 Agenda for Sustainable Development’ called for international action to combat viral hepatitis, with the aim of drastically reducing the disease burden by 2030. One of the barriers to achieving this goal is incomplete local surveillance systems [14].

The main strength of this analysis is that for the first time, it covered whole cases reported via the national Lithuanian surveillance system during a 14-year period. To the co-authors’ knowledge, an analysis of acute HCV infection cases in Lithuania of this magnitude has not been published previously. The Limitations of this research include the following:

It is likely that a large number of acute HCV infection cases were not included in this analysis because they were not captured by the current surveillance system. Newly acquired HCV infections are mainly asymptomatic, and these infected people do not seek medical help. It is worth mentioning that the proportion of symptomatic cases among new cases is likely to be constant; the epidemiological patterns described in this analysis are therefore likely to be accurate.

Only acute cases of HCV infection were included in this study, because chronic cases of HCV infection only began to be recorded by the Lithuanian national surveillance system in 2019.

Potential exposure factors were evaluated based on information provided by the subject, which may have led to several biases (a person may forget things that are not important to them, or they might want to hide sensitive, personal information, and so on).

Conclusions regarding potential exposure sources for acute HCV infection are greatly limited by the fact that, for a large proportion of the cases, information regarding the route of infection was unknown or missing.

Due to the limitations of this study mentioned above, the data obtained cannot be assumed to be exact, but it can illustrate the approximate magnitude and risk groups of the problem. A decreasing IR trend of acute HCV cases and the limited identification of potential exposures can be a warning indicator of actual HCV spread in Lithuania. The large proportion of HCV infections remain asymptomatic, and the real risk could be devalued, especially when taking into account the fact that chronic cases of HCV only started to be recorded in Lithuania in 2019. Even the WHO recognised viral hepatitis as a ‘largely ignored’ health problem, which led to a World Health Assembly resolution to eliminate viral hepatitis as a major public health problem by 2030 [15]. The world now has the tools to cure hepatitis C—new, direct-acting antivirals which can cure 95% of chronic infections—though these drugs are unlikely to reach all chronically infected people. The key to the elimination of hepatitis, then, is the identification of people who have the disease and ensuring that they are appropriately provided withcare. One of the biggest challenges in fighting viral hepatitis is therefore the improvement of national screening strategies.

## 5. Conclusions

Being male, aged between 25 and 34 years, and living in the city might be important factors for understanding HCV epidemiological patterns in Lithuania. However, only 43.8% of the subjects included in this analysis reported the potential exposure source. This shows that estimations of the risk factors should be improved with detailed case investigations. Even though, the number of HCV cases diagnosed and registered in Lithuania showed a decreasing trend, our country has one of the highest IRs when compared to other European countries. Bearing in mind that most asymptomatic HCV cases are not represented in official statistics, additional sources of data regarding HCV infection are needed. Lithuania should choose a cost-effective screening method for HCV, which can lead to the implementation of a national screening programme.

## Figures and Tables

**Table 1:** Yearly changes of incidence of acute hepatitis C virus infection in different age groups, Lithuania, 2005–2018.

Age Group	Exp(B)	95% CI	*p*	Yearly Change, %
0–24	0.87	0.83–0.91	<0.0001	−13.1
25–54	0.93	0.90–0.96	<0.0001	−7.2
55–74	0.96	0.90–1.02	0.179	−4.0
75+	0.89	0.78–1.01	0.064	−11.2

**Table 2:** Cases of acute HCV infection reported in Lithuania and Europe, 2012–2018.

Year	Lithuania		EU/EEA ^1^	
	N	Rate ^2^	N	Rate ^2^
2012	40.0	1.3	509.9	0.6
2013	59.0	2.0	569.0	0.5
2014	34.0	1.2	458.0	05
2015	23.0	0.8	346.0	0.4
2016	16.0	0.6	813.0	0.4
2017	25.0	0.9	861.0	0.3
2018	25.0	0.9	1325	0.4

Absolute numbers and rates of diagnosed and reported acute HCV cases per 100,000 people in Lithuania and the European Union/European Economic Area countries from 2012 to 2018 years. EU/EEA*—*European Union/European Economic Area countries. N—absolute number of cases. ^1^ Absolute numbers and rates for the HCV cases diagnosed in the EU/EEA were collected from the annual surveillance reports prepared by the European Centre for Disease Prevention and Control for the period from 2012 to 2017. ^2^ Notification rates were calculated per 100,000 members of the population annually.

## Data Availability

The statistical reporting forms published monthly and annually by the Centre for Communicable Diseases and AIDS are available online at http://www.ulac.lt/ataskaitos.
